# From nociception to pain perception, possible implications of astrocytes

**DOI:** 10.3389/fncel.2022.972827

**Published:** 2022-09-07

**Authors:** Frida Higinio-Rodríguez, Angélica Rivera-Villaseñor, Isnarhazni Calero-Vargas, Mónica López-Hidalgo

**Affiliations:** ^1^Escuela Nacional de Estudios Superiores, Universidad Nacional Autónoma de México, Querétaro, Mexico; ^2^Instituto de Neurobiología, Universidad Nacional Autónoma de México, Querétaro, Mexico

**Keywords:** astrocytes, sensory, nociception, perception, pain, calcium activity

## Abstract

Astrocytes are determinants for the functioning of the CNS. They respond to neuronal activity with calcium increases and can in turn modulate synaptic transmission, brain plasticity as well as cognitive processes. Astrocytes display sensory-evoked calcium responses in different brain structures related to the discriminative system of most sensory modalities. In particular, noxious stimulation evoked calcium responses in astrocytes in the spinal cord, the hippocampus, and the somatosensory cortex. However, it is not clear if astrocytes are involved in pain. Pain is a private, personal, and complex experience that warns us about potential tissue damage. It is a perception that is not linearly associated with the amount of tissue damage or nociception; instead, it is constructed with sensory, cognitive, and affective components and depends on our previous experiences. However, it is not fully understood how pain is created from nociception. In this perspective article, we provide an overview of the mechanisms and neuronal networks that underlie the perception of pain. Then we proposed that coherent activity of astrocytes in the spinal cord and pain-related brain areas could be important in binding sensory, affective, and cognitive information on a slower time scale.

## Introduction

### From nociception to pain

According to the International Association for the Study of Pain (IASP), pain is defined as an unpleasant sensory and emotional experience associated with, or resembling that associated with, actual or potential tissue damage (Raja et al., 2020). It is a complex experience defined by biological, psychological, and social factors with sensorial, emotional, and cognitive components. It is important to make clear that pain and nociception are not the same phenomena; nociception is related to the activation, transfer, and processing of noxious stimuli within the CNS (Loeser and Treede, 2008). Instead, pain is an individual experience that, in physiological conditions, warns us about potential or real tissue damage. Although pain can be induced by different modalities, here we will focus on nociception and pain evoked by the somatosensory system.

When you harm yourself, the noxious stimulus is detected in the periphery by receptors called nociceptors. This information arrives at the dorsal horn of the spinal cord (Dubin and Patapoutian, 2010) and is transmitted to the thalamus directly through the spinothalamic tract or indirectly through the spinoreticular and spinomesencephalic tracts (Giesler et al., 1981; Willis and Westlund, 1997). From the canonical view, thalamic neurons from the ventral posterolateral (VPL) and ventral posteromedial nucleus (VPM) relay nociceptive information from the body and the head, respectively, to the primary somatosensory cortex (SI) to process sensory components such as quality, intensity, and location of nociceptive stimuli (Backonja, 1996; Monconduit et al., 2006; Kim et al., 2019). From there, the information spread to hierarchically higher cortical regions to transform nociception into pain perception.

**FIGURE 1:**
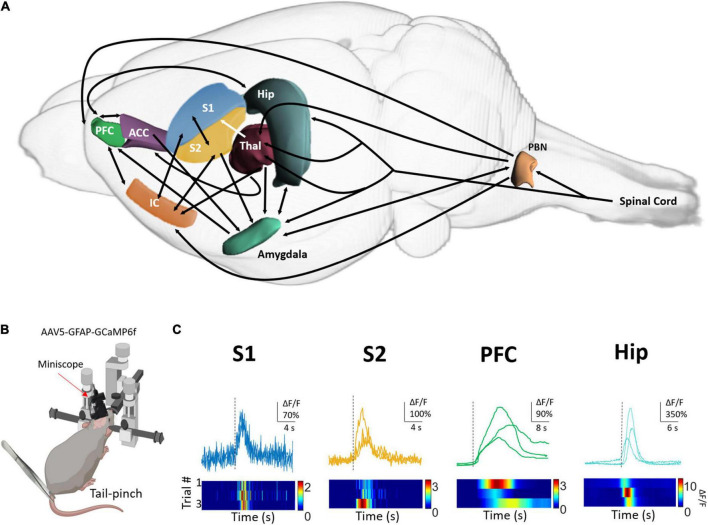
Astrocytes respond to tail-pinched stimulation with calcium increases in pain-related brain areas. **(A)** Diagram showing regions (in color) and connections (arrows) of the nervous system that process nociceptive stimuli and are involved in pain perception. PFC, prefrontal cortex; ACC, anterior cingulate area; Hip, Hippocampus; S2, secondary somatosensory cortex; S1, primary somatosensory cortex; IC, insular cortex; Thal, thalamus. **(B)** Tail-pinch was applied with forceps to lightly anesthetized mice (0.5% isoflurane) expressing GCaMP6f in astrocytes located in S1, S2, PFC, and Hip. Representative traces of astrocyte calcium dynamics were monitored with one-photon Miniscope. Tail-pinch stimulation is indicated with a dotted black line **(C)**. The color maps show the calcium responses before, during, and after tail pinch stimulation. Basal fluorescence was considered as the calcium activity observed before the sensory stimulation.

However, growing evidence shows that nociceptive information is processed in parallel in multiple brain areas (Figure 1A) and that is necessary to bind sensory, affective, and cognitive components to generate the experience of pain (Coghill, 2020). The thalamus, more than a sensory relay, is a key brain structure interplaying cortical and subcortical brain structures to integrate the components of the pain experience. The thalamic nuclei that directly received nociceptive information from the periphery and relay it to the primary sensory cortex are classified as first-order (VPL and VPM); high-order thalamic nucleus (i.e., posterior nucleus) receive information from the deep cortical layer from S1 and send it back to S1, secondary somatosensory cortex (S2), and primary motor cortex to modulate the motor response to pain (Liao and Yen, 2008; Zhang and Bruno, 2019). The thalamus is also connected with the amygdala, the anterior cingulate, and the insular cortex, brain structures involved in the affective component of pain; it also projects to the association cortices such as the prefrontal and the parietal cortices related to the cognitive component of pain (Petrovic et al., 2000; Yen and Lu, 2013). This is relevant because although several brain structures are active during nociception, the activity of these structures by themselves cannot explain the pain experience, instead, the perception of pain requires the coordinated activity from many of these brain regions, and the thalamus is well located to accomplish this function.

The parabrachial nucleus (PBN) receives direct information from nociceptive neurons located in the superficial lamina of the dorsal horn of the spinal cord through the spinoparabrachial tract. It processes visceral pain due to noxious thermal stimuli and inflammatory process (Willis, 1985; Bester et al., 1997) and conveys several types of information such as a taste, pain, and aspects of autonomic control like respiration, blood pressure, fluid balance, and thermoregulation (Chiang et al., 2020). It has reciprocal connections with the central nucleus of the amygdala, the nucleus of the bed of the stria terminalis, and hypothalamic nuclei. The PBN have an important role in the autonomic, motivational and affective responses to pain (Schaible and Grubb, 1993; Millan, 1999) regulating the emotional and autonomic aspects of pain experience (Bourgeais et al., 2001).

In S1, the neuronal activity is somatotopically organized (Mancini et al., 2012) providing information about the location of the noxious stimulation (Jin et al., 2018). Its neuronal activity correlates with the properties of the stimulus including the intensity (Kenshalo et al., 2000; Bufalari et al., 2007; Iwamoto et al., 2021). These neurons are highly modulated by prior experiences or cognitive factors such as attention. For example, when a subject is diverted from a painful stimulus, the nociceptive-evoked neuronal responses are reduced (Backonja, 1996; Bushnell et al., 1999). On the other side, neurons from S2 respond bilaterally to the noxious stimuli (Davis et al., 1998) coding its intensity with increases in neuronal activity. Although this region is also involved in nociceptive processing (Davis et al., 1998; Coghill et al., 1999), the activation of S2 is better associated with the scores of a sensory discriminative component induced by different pain modalities (Maihöfner et al., 2006).

The emotional component of pain is related to the unpleasantness of the nociceptive stimuli. This is by definition, an essential component for the categorization of an experience as painful. It can be associated with the stimulus intensity (Wilcox et al., 2015), but also be independent of it and related to individual, contextual, or cultural aspects. Some of the brain areas involved in this pain-related affective system include the anterior cingulate cortex (Tölle et al., 1999), the amygdala (Straube et al., 2011), the insula (Kim et al., 2017), and the hippocampus (Angenstein et al., 2013).

The basolateral amygdala receives sensory information from the thalamus and cortical areas (LeDoux et al., 1990; Shi and Davis, 1999) whereas the central amygdala receives nociceptive information directly from the spinal cord (Cliffer et al., 1991; Burstein and Potrebic, 1993) and parabrachial area (Bernard and Besson, 1990; Gauriau and Bernard, 2002). The anterior cingulate cortex is connected to the thalamus, prefrontal cortex (orbitofrontal and the medial portion), and amygdala. They mediate fight behaviors in response to noxious stimuli (Gao et al., 2004). The cingulate and the prefrontal cortex are also active when the subject perceives pain from others and has been linked to prosocial behavior and empathy (Kim et al., 2021).

The hippocampus receives nociceptive inputs from the periphery through the spinothalamic and parabrachial pathways and directly from the spinal cord through the septo-hippocampal (Mokhtari et al., 2019). It works together with the anterior cingulate, the insula, the amygdala, the nucleus accumbens, and the prefrontal cortex (Thompson and Neugebauer, 2019) to combine emotional and cognitive aspects of pain perception. For example, remembering a painful moment can induce pain by itself (Babel, 2017), the novelty of a painful stimulus is determinant for the percept of pain (a process known as a “pain alarm”). The importance of the activity of this pain matrix is also relevant during the expectancy-based modulation of pain in placebo and hypnosis-induced analgesia (Bushnell et al., 2013; Geuter et al., 2013; Keller et al., 2018), as well as during “pain catastrophizing,” where the subject tends to magnify a possible threat of painful stimulus, with constant ruminant thoughts associated with pain anxiety and helpless (Quartana et al., 2009).

Individual pain experiences depend on the activation of regions and networks that are spatially distributed through the brain that engage during nociception (Kim and Davis, 2020) commonly referred to as “pain matrix.” The integration of pain components depends on the synchronization of neuronal activity in parallel but also complex temporal patterns of brain activity to allow communication in a long-range scale (Hauck et al., 2008; Barardi et al., 2014; Peng and Tang, 2016). Binding oscillatory activity, in particular gamma oscillations, in the pain matrix has been proposed as the mechanism to integrate these components to generate a complete perceptual representation of pain (Sedley and Cunningham, 2013; Ghiani et al., 2021). In particular, nociceptive stimulation can induce by itself, coherent gamma activity (Tan et al., 2019) in sensory (Gross et al., 2007; Kim et al., 2015) and affective-cognitive pathways (Hauck et al., 2017; Liberati et al., 2018; Xiao et al., 2019), that have a direct relation with pain ratings in humans (Ploner et al., 2017; May et al., 2019). Although the neural mechanism related to the generation of gamma oscillations is still under investigation, it has been proposed that the activity of fast-spiking inhibitory interneurons, and neuromodulator systems are essential for gamma rhythm (Kim and Davis, 2020).

### Astrocytes respond to nociceptive stimulation

Astrocytes are electrically non-excitable cells that exhibit changes in cytosolic calcium concentration as a form of excitability in response to neuronal activity (Parpura and Verkhratsky, 2012; Zorec et al., 2012; Bazargani and Attwell, 2016). Calcium events are observed throughout the astrocyte due to the activation of ionotropic calcium-permeable receptors or metabotropic receptors linked to phospholipase C/inositol trisphosphate receptors (IP_3_R) (Foskett et al., 2007; Shigetomi et al., 2012; Okubo, 2020; Sherwood et al., 2021). Astrocyte-calcium events are diverse in terms of the spatial extension, they can be localized to some subcellular domains (López-Hidalgo et al., 2017), extended to the entire astrocyte or throughout gap junctions forming an astrocyte network (Hirase et al., 2004; Hoogland et al., 2009).

Astrocytes respond to different sensory modalities in cortical and subcortical regions involved in sensory processing (Figures 1B,C) such as the spinal cord, olfactory bulb, visual, auditory, and somatosensory cortex (Petzold et al., 2008; Schummers et al., 2008; Ghosh et al., 2013; Otsu et al., 2015; Sekiguchi et al., 2016; Lopez-Hidalgo et al., 2019; Lines et al., 2020). In these structures, evoked-calcium responses encode the intrinsic properties of the stimulus such as duration, intensity, location, and modality and share the topographical organization of neuronal maps (Ghosh et al., 2013; López-Hidalgo and Schummers, 2014).

In the somatosensory system, astrocytes respond to tactile and noxious stimuli in regions related to nociception and the perception of pain. In a seminal work by Sekiguchi et al. (2016), sensory-evoked calcium activity of dorsal horn astrocytes was not correlated with the duration and intensity of the mechanical stimulus whereas the underlying neuronal activity was positively correlated suggesting that astrocyte activity is not secondary to the activation of neurons of the pain matrix. Astrocytes respond to low and medium pressure-amplitude applied to the tail of freely moving mice, with increases in the frequency of astrocyte calcium events, however, this activity was mostly restricted within the astrocytes. On the other side, high mechanical pressure induced large-scale synchronized calcium activity in astrocytes but not in neurons. Although it is not clear if non-nociceptive or nociceptive fibers are activated during low/medium or high pressures, respectively, this suggests that somatosensory-evoked calcium responses on astrocytes depend on the sensory modality more than the stimulus intensity (Sekiguchi et al., 2016).

In the somatosensory cortex, astrocyte calcium responses encode different parameters of the somatosensory stimuli (Wang et al., 2006; Winship et al., 2007; Stobart et al., 2018; Lines et al., 2020). The stimulation of the peripheral receptive field of S1 (Winship et al., 2007; Stobart et al., 2018) and barrel cortex induced evoked calcium responses in astrocytes that depend on the intensity and the frequency of the stimulus (Wang et al., 2006; Thrane et al., 2012; Stobart et al., 2018). However, as occurs in the spinal cord, low-intensity electrical stimulation (0.4–0.6 mA) induces spatially restricted calcium responses within the astrocytes that do not extend in the field of view (Ghosh et al., 2013; Zhang et al., 2016). However, high-intensity electrical stimulation (1–3 mA) evoked large-scale calcium responses in astrocytes (Gu et al., 2018; Lines et al., 2020) which reinforces the idea that nociceptive information recruited astrocytes networks.

Noxious stimulation (footshock) can induce reliable calcium activity in astrocytes in other brain areas related to pain perception such as the hippocampus (Zhang et al., 2021). Moreover, auditory cortical astrocytes are more responsive to footshock (67%) in comparison to the number of astrocytes activated by the natural sensory stimuli, sound (8%). Here, astrocytes respond with a coordinated large-scale activity that is partially mediated by gap junctions and depends on the nicotinic acetylcholine receptors (Zhang et al., 2021). This supports the relevant role of neuromodulatory systems in mediating nociceptive-evoked responses in astrocytes throughout the nervous system. Furthermore, this evidence highlights the importance of nociception in shaping the activity of brain cortical circuits and hence the behavior. In this case, Zhang et al. (2021) showed that footshock-evoked calcium activity in auditory cortical astrocytes is induced in fear memory and its extinction goes in parallel with the extinction of the behavior.

Data obtained in our laboratory from astrocytes expressing GCaMP6f in anesthetized mice (Figure 1B) extend the previous evidence regarding the nociceptive-evoked calcium responses (tail pinch) in pain-related areas. Here, we provide evidence that astrocytes located in S2 and prefrontal cortex also respond to nociceptive stimulation as occurs in astrocytes from S1 and hippocampus (Figure 1C). Although there are differences in the amplitude, the delay, and the duration of nociceptive-evoked calcium responses in astrocytes, one common characteristic among these regions is that nociceptive stimulation recruited a large portion of astrocytes (Gu et al., 2018; Lines et al., 2020). In this context, it is tempting to propose that nociceptive stimuli activate coherent activity of astrocytes located in pain-related brain areas acting as an “astrocyte pain matrix” that in conjunction with the neural activity would construct pain perception. Global widespread activation of astrocytes can be induced by the increase in glutamate and K^+^ levels in the synaptic space due to synchronic neuronal activity (De Pittà et al., 2008). The activation of astrocytes can lead to gliotransmitter release acting as a paracrine signal to activate nearby astrocytes generating a “domino effect” to spread calcium signaling in large-scale proportions (Pereira and Furlan, 2009; Goldberg et al., 2010; Lallouette et al., 2019). Another possibility is that global astrocyte activity is not secondary to the activation of neurons, instead, nociceptive stimulation could open gap junctions located in the astrocytes to allow the activation of the syncytium. In fact, connexin 30, 43, and 32 are highly expressed in pain-related brain areas such as the spinal cord, thalamus, S1, prefrontal cortex, and cingulate cortex (Hirase et al., 2004; Houades et al., 2008; Ernst et al., 2011; Zhang et al., 2013; Fujii et al., 2017). In any case, the activity of the coordinated astrocytes could in turn feeds back to the neuronal circuit (Pereira et al., 2013) increasing the synchronization (Makovkin et al., 2022) and hence contributing to the binding of the information and pain perception.

### Perspective

Cutaneous tactile stimulation evoked sparser calcium activity in astrocytes (Stobart et al., 2018). This favors local neuron-astrocyte interaction facilitating the location of the stimulus in places with a topographic organization. Instead, nociception informs the nervous system of real or potential damage and produces pain to protect the harmed area. In this scenario, the cortical representation of the location of the stimulus extends to nearby areas developing a state of hyperalgesia around the damaged zone to ensure the protection of the area while the integrity of the tissue is restored. In this context, global astrocytes activity in the cortex could be involved in short and long-term plasticity to ensure this state of hyperalgesia.

Although nociceptive processing occurs unconsciously, emotional, socio-cultural, and cognitive factors (such as attention) are relevant in producing pain perception; therefore, a state of consciousness is necessary to construct the experience of pain. However, an important question in neuroscience that remains to be answered is how pain is created from nociception? How are the elements that compose the percept of pain binding to provide a painful experience? Here, we provide evidence about global and synchronous calcium activity in astrocytes evoked by nociceptive stimulation in areas related to the pain experience. It seems plausible that a coherent activity of astrocytes in the brain and the spinal cord could be important to bind sensory, affective, and cognitive information on a slower time scale forming an astrocyte pain matrix. According to this, Pereira and Furlan (2010) proposed that individual astrocytes could operate as a “local hub” integrating information within their local domain. However, the astrocyte pain matrix would communicate astrocytes within the pain network (Pereira and Furlan, 2010; Lallouette et al., 2014) acting as a “Master Hub” integrating information from several brain areas that could be involved in the process of perception as occurs with pain. Furthermore, because astrocytes can in turn increase the synchronization of neuronal networks (Makovkin et al., 2022), it could be involved in directly modulating gamma activity and hence in the binding process. Another possibility is that astrocytes respond directly to neuromodulators such as acetylcholine, norepinephrine, and dopamine (Jennings et al., 2017; Covelo and Araque, 2018) that are released by nociceptive stimulation (Wahis and Holt, 2021) resulting in changes in the excitability and synchrony of neural networks (Sardinha et al., 2017; Adamsky et al., 2018; Bellot-Saez et al., 2018) that are necessary for the perception of pain.

Brain oscillations in the frequencies of the gamma band have been linked to the perception of pain (Tan et al., 2019; Kim and Davis, 2020). These oscillations are present throughout the brain (Buzsáki and Draguhn, 2004) including regions such as the sensory, prefrontal, insula, and anterior cingulate cortex (Gross et al., 2007; Kim et al., 2015; Hauck et al., 2017; Liberati et al., 2018; Xiao et al., 2019). In 2020, Lines and colleagues showed that sensory stimulation in the paw induces an increase in gamma activity in the primary somatosensory cortex that correlates with the intensity of the stimulus (Lines et al., 2020). In parallel, sensory stimulation induces global calcium responses in cortical astrocytes with a delay in the order of seconds. Moreover, the manipulation of calcium activity in astrocytes was inversely correlated with gamma activity demonstrating that activation of astrocytes with DREADDs is sufficient to decrease gamma oscillations. On the other side, Lee et al. (2014) observed during a spatial memory task (Y maze), that calcium elevations in hippocampal astrocytes precede the onset of gamma activity in the hippocampus. This could imply a different role of astrocytes regulating gamma activity depending on the level of arousal (anesthetized vs. awake), brain structures (somatosensory cortex vs. hippocampus) as well as underlying neural activity (natural activity in behaving animal vs. sensory stimulation).

High-order thalamic nucleus plays an important role in making consciousness of an experience thanks to the connections with cortical and subcortical regions, where it acts as a sensory activity filter and synchronized neuronal activity (Ward, 2011). Although it is unknown if thalamic astrocytes respond to nociceptive stimulation with calcium activity and the extension of these responses, thalamic astrocytes regulate the sensory acuity of mice in a tactile-discriminatory task by releasing GABA (Kwak et al., 2020). In this scenario, it would be interesting to analyze if GABA release by astrocytes could set an inhibitory tone that regulates gamma activity as occurs with inhibitory interneurons and hence modulate gamma activity and its impact on perceptions.

There are still fundamental questions in neuroscience that are still poorly understood despite all the technical advances and progress in neurobiology such as the process of creating perception from sensations. Thus, is tempting to propose that astrocytes are well equipped, located, and connected to regulate, in parallel, multiple neuronal networks allowing individual pain experiences. However, further work integrating astrocytes in this field is required.

## Data availability statement

The raw data supporting the conclusions of this article will be made available by the authors, without undue reservation.

## Ethics statement

This animal study was reviewed and approved by Instituto de Neurobiología at Universidad Nacional Autónoma de México (No. 043).

## Author contributions

All authors drafted, edited, and approved the final version of the manuscript.
